# Supplementary material for: Surface diffusion-limited lifetime of silver and copper nanofilaments in resistive switching devices
Source: Nat Commun. 2019 Jan 8;10:81. doi: 10.1038/s41467-018-07979-0 (PMC6325242; doi:10.1038/s41467-018-07979-0)
Supplement: Supplementary file 1 — Supplementary Information [file 41467_2018_7979_MOESM1_ESM.docx]

**Supplementary Information for**

# Surface diffusion-limited lifetime of silver and copper nanofilaments in resistive switching devices

Wang et al.

**
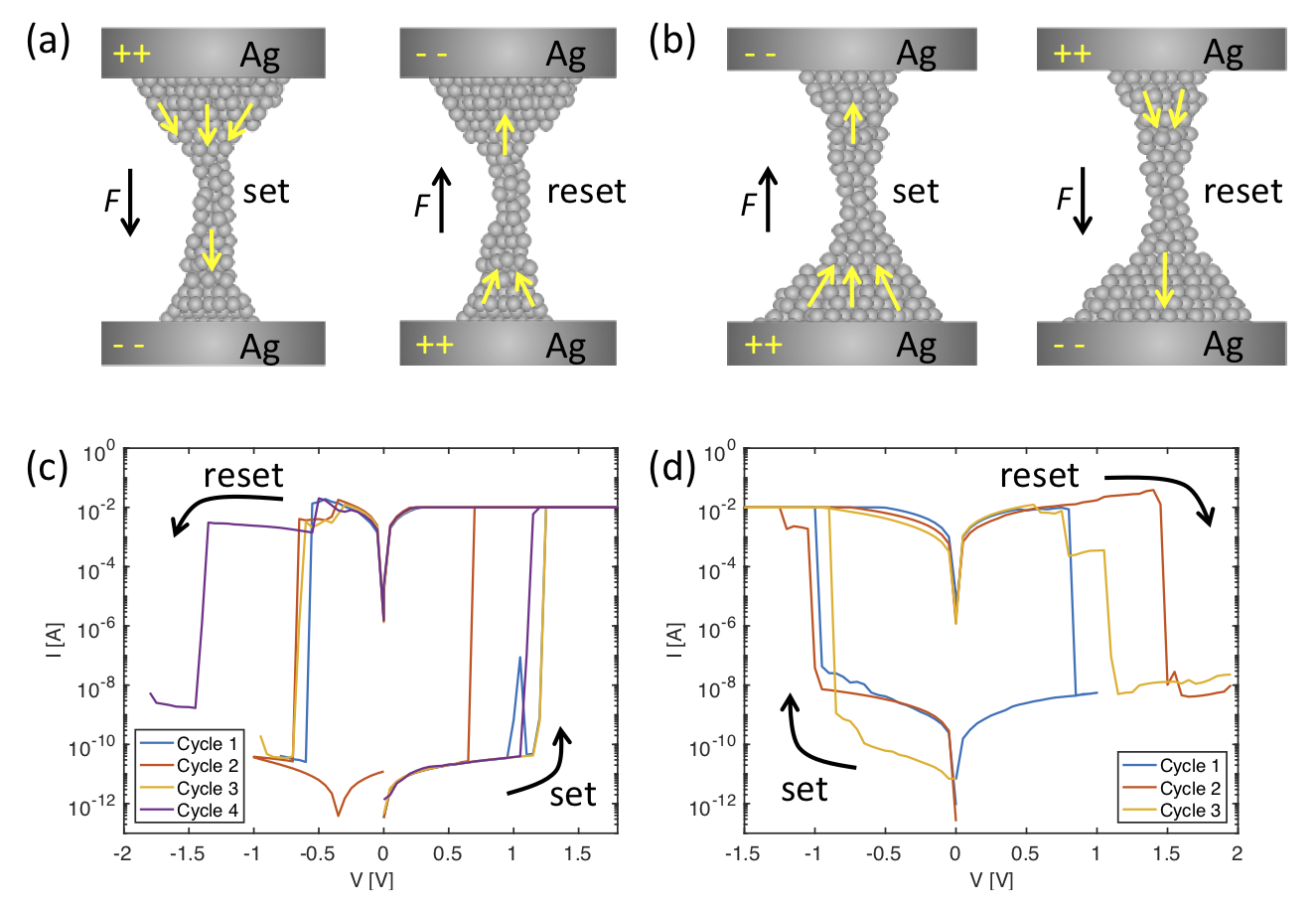
**

**Supplementary Figure 1. Bidirectional bipolar switching characteristics.** Atoms injected from one of the two electrodes give rise to the filament (a, b). Thanks to the symmetric device structure, with Ag electrodes at both sides of the insulating layer, non-volatile switching can take place either by set transition under positive voltage and reset transition under negative voltage (c) or by set transition under negative voltage and reset transition under positive voltage (d).

**
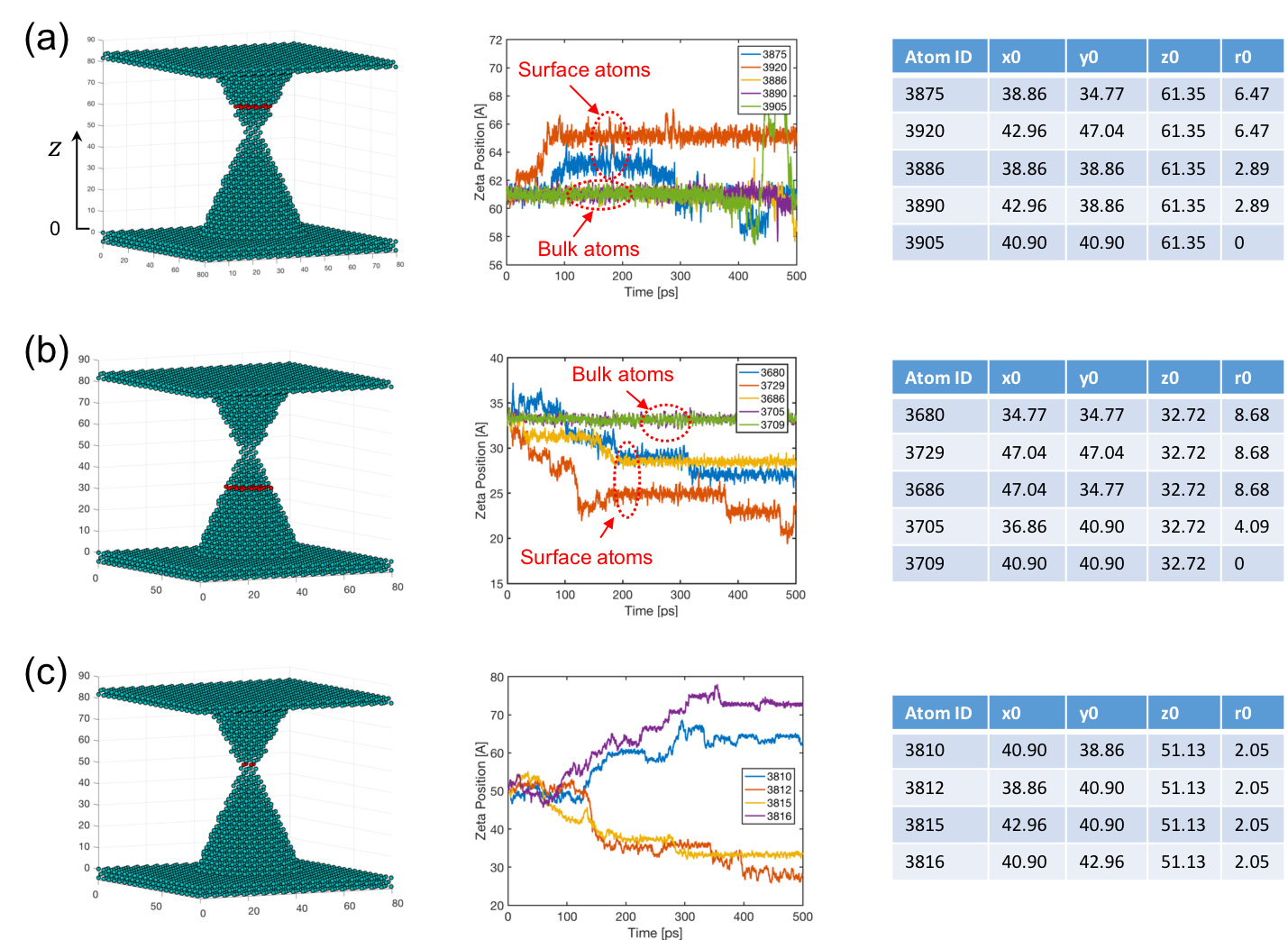
**

**Supplementary Figure 2.** **MD simulations of the surface self-diffusion effect in a nanoscale Ag filament by tracking of individual atoms at various positions along the filament.** (a) Position of individual atoms close to the top electrode (left), simulated atom traces along the z direction (center), initial positions (x0, y0, z0) and horizontal distance from the filament center (r0) of the traced atoms (right). (b) Same as (a), but for atoms close to the bottom electrode. (c) same as (a) and (b), but for atoms in the bottleneck region of the filament. All space coordinates are reported in Å.


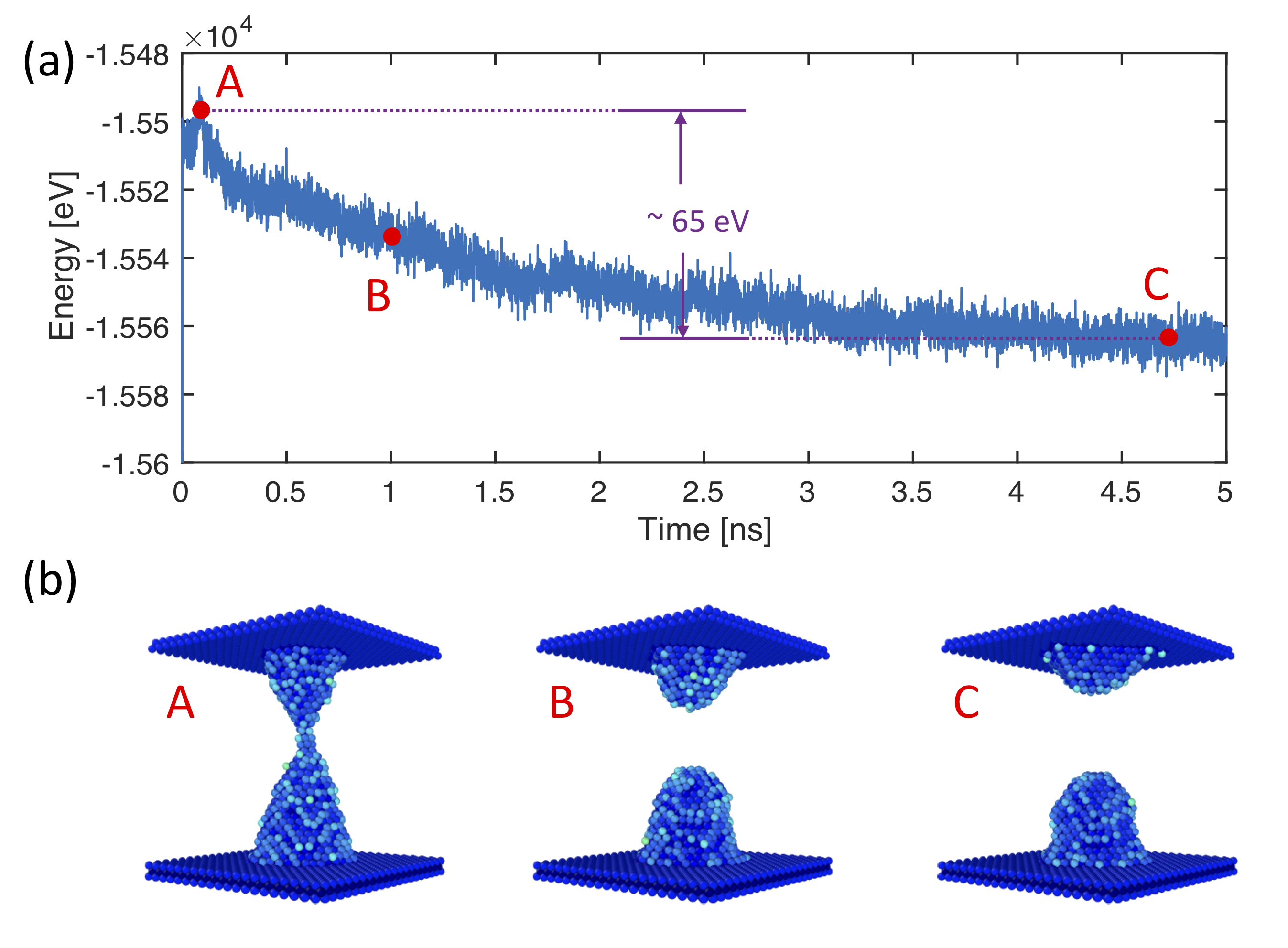


**Supplementary Figure 3.** **Energy relaxation of the MD simulated metal filament.** (a) Total energy as a function of simulation time. (b) Corresponding filament shapes for points A, B, and C in (a).

**
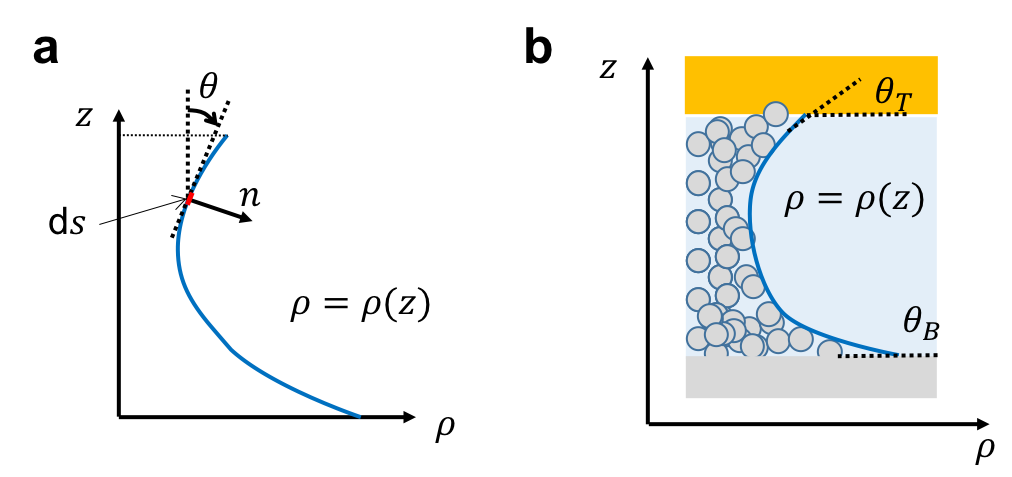
**

**Supplementary Figure 4. Numerical calculation details.** (a) Calculation details of the morphological evolutions. (b) Illustration sketch for the boundary condition defined by top and bottom electrodes.


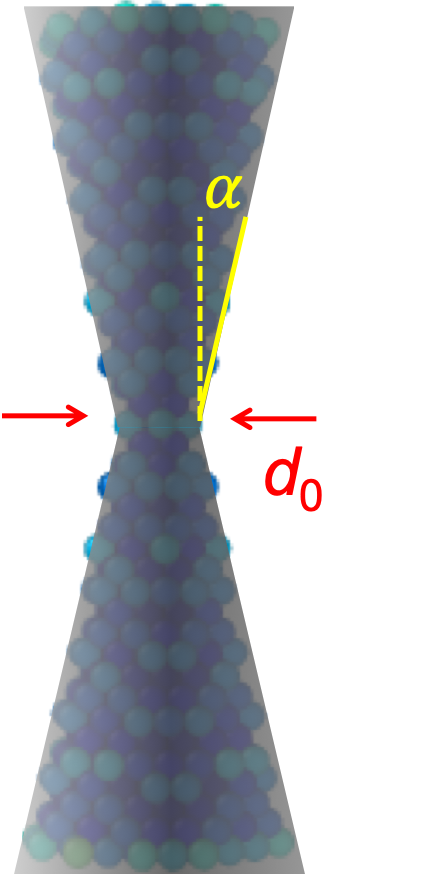


**Supplementary Figure 5.** **Initial filament shape.** Illustration of the initial filament geometry, where the CF is assumed to consist of two truncated cones in back-to-back configuration.


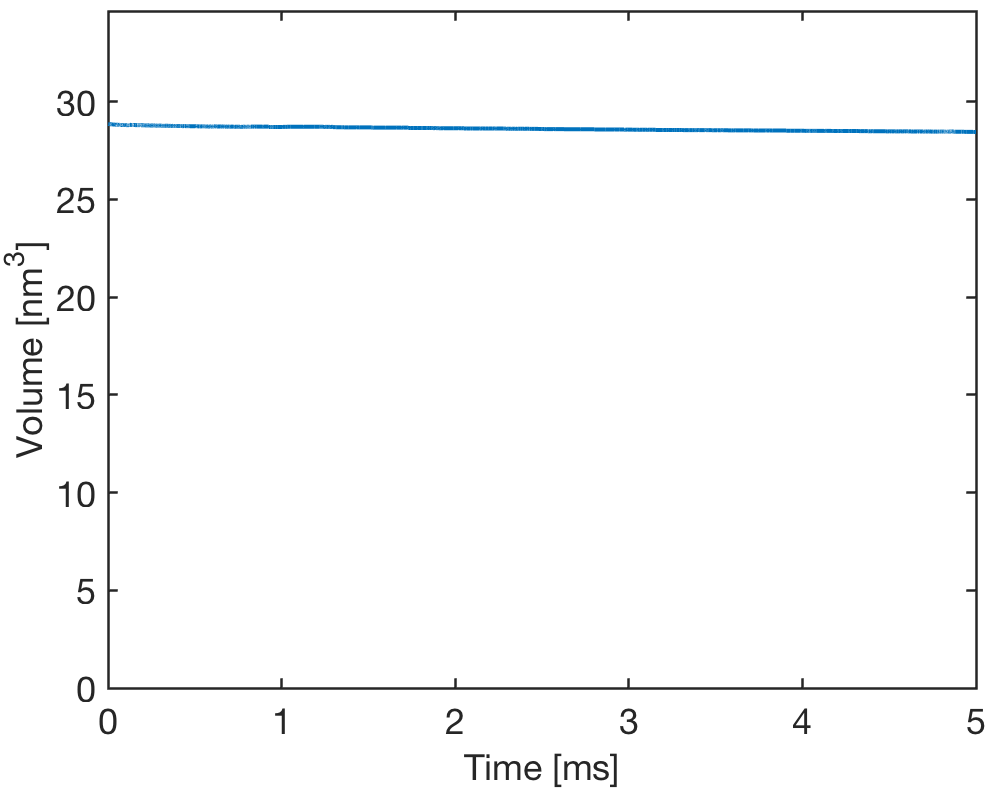


**Supplementary Figure 6.** **Recorded volume during the filament morphological evolution**.

**
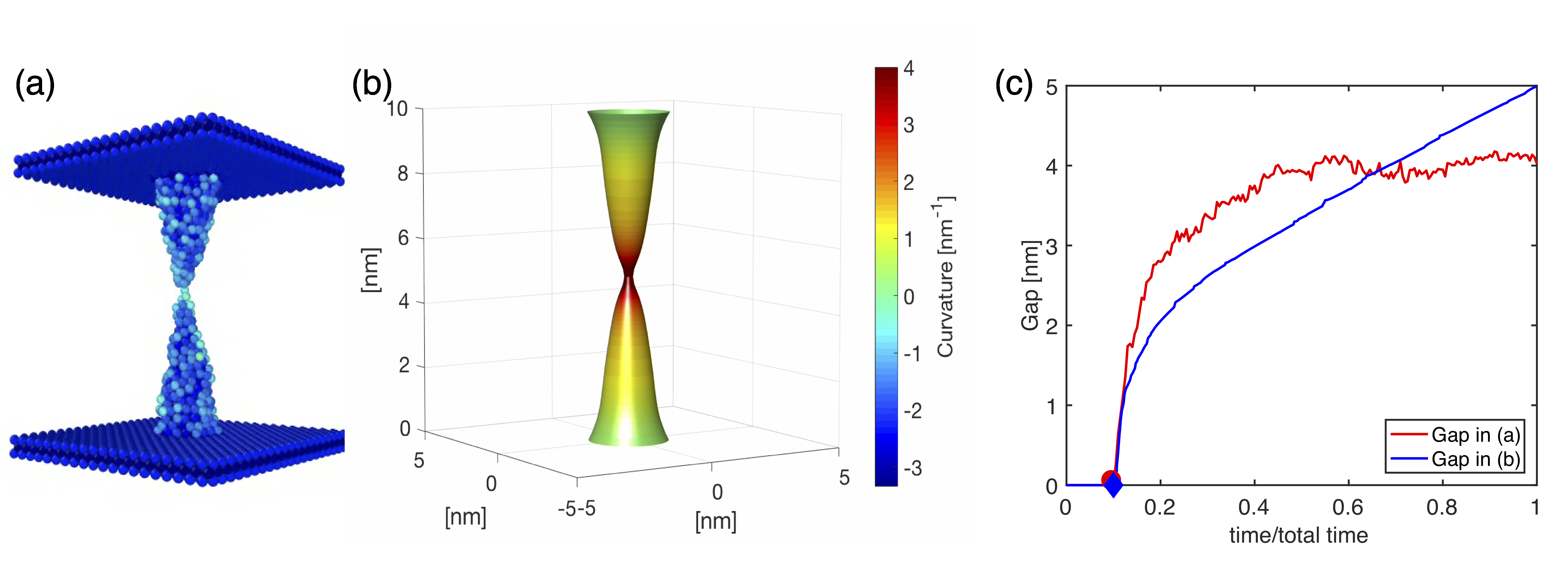
**

**Supplementary Figure 7.** **Comparison of the MD simulation and the numerical model for the filament relaxation process**. (a) MD simulation of a filament relaxation process. (b) Numerical simulation based on surface diffusion mechanism of a similar filament as the one in (a). (c) Filament gap length as a function of normalized time.


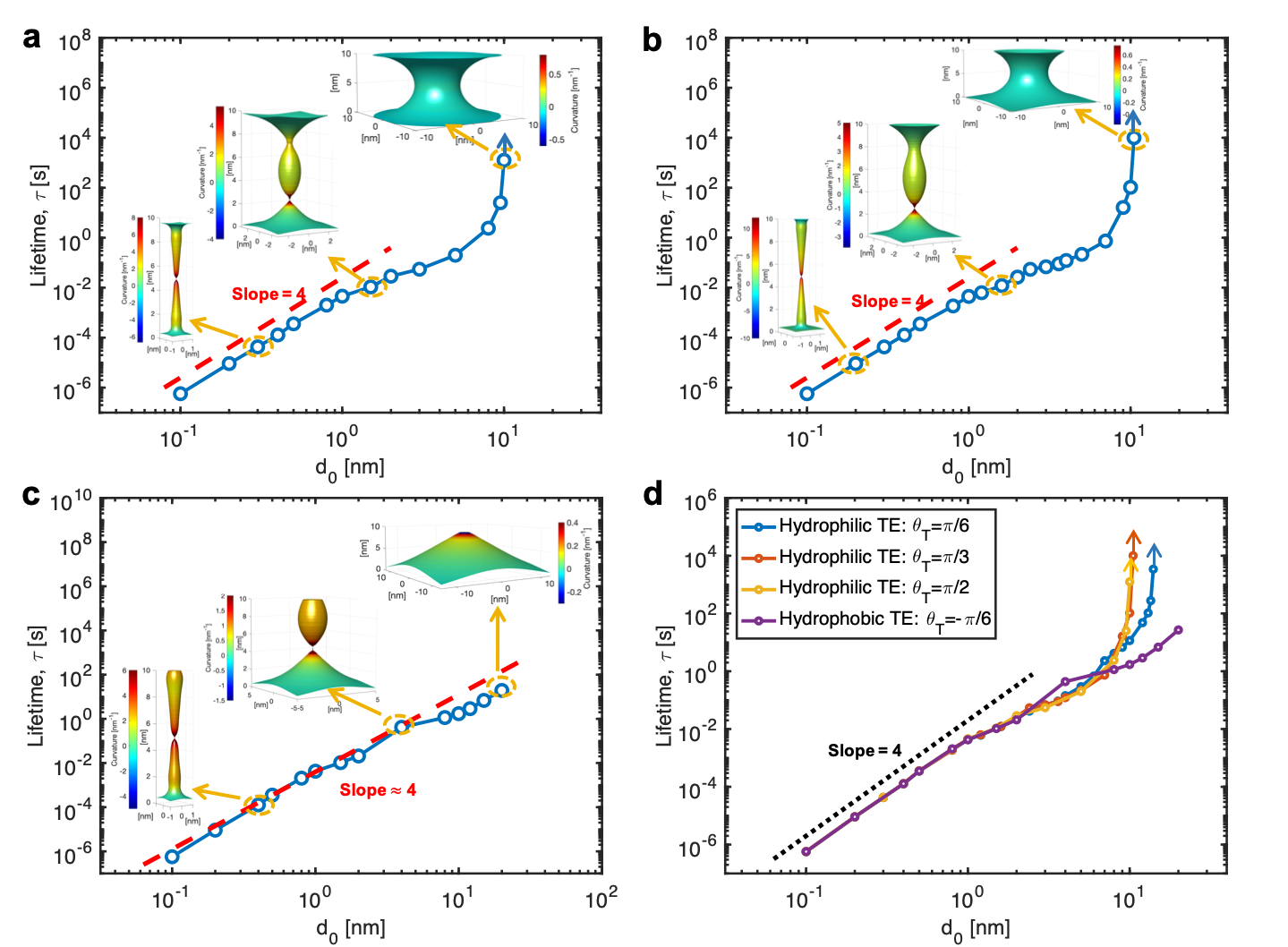


**Supplementary Figure 8.** **Filament lifetime as a function of the normalized diameter for various electrode materials.** (a) Filament lifetime as a function of the initial filament diameter for a filament length of $h=10 \mathrm{nm}$. The boundary conditions are $\theta_{T}=\pi/2$ and $\theta_{B}=-\pi/2$, i.e., both the top and the bottom electrodes are made of the same material as the filament. (b) Same with (a) except the boundary conditions of $\theta_{T}=\pi/3$ and $\theta_{B}=-\pi/2$, corresponding to different top/bottom electrode materials, where the top electrode is made of the same material as the filament. In both figures (a) and (b), the last point at $d_{0}/h\approx1$ in the figures shows a lower boundary of lifetime, as the maximum simulation time was about 10^4^ s. (c) Same with (a) except the boundary conditions of $\theta_{T}=-\pi/6$ (i.e., the top electrode is hydrophobic with respect to the filament material) and $\theta_{B}=-\pi/2$ (i.e., the bottom electrode is made of the same material as the filament material). (d) Calculated filament lifetime for various top electrode boundary conditions, i.e., various top electrode materials.

**
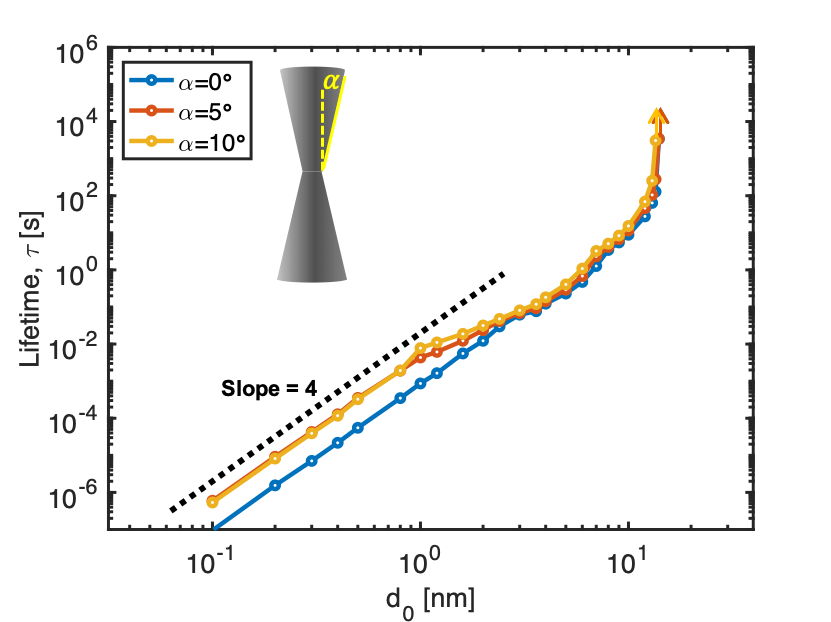
**

**Supplementary Figure 9.** **Calculated filament lifetime as a function of initial diameter for various initial filament shapes.** The lifetime was calculated as a function of the initial diameter for various initial filament shapes, namely a conical shape (see the inset) with various conical angle$\alpha=0^{\circ}$, $\alpha=5^{\circ}$ and$\alpha={10}^{\circ}$ (filament length $h=10 \mathrm{nm}$.).


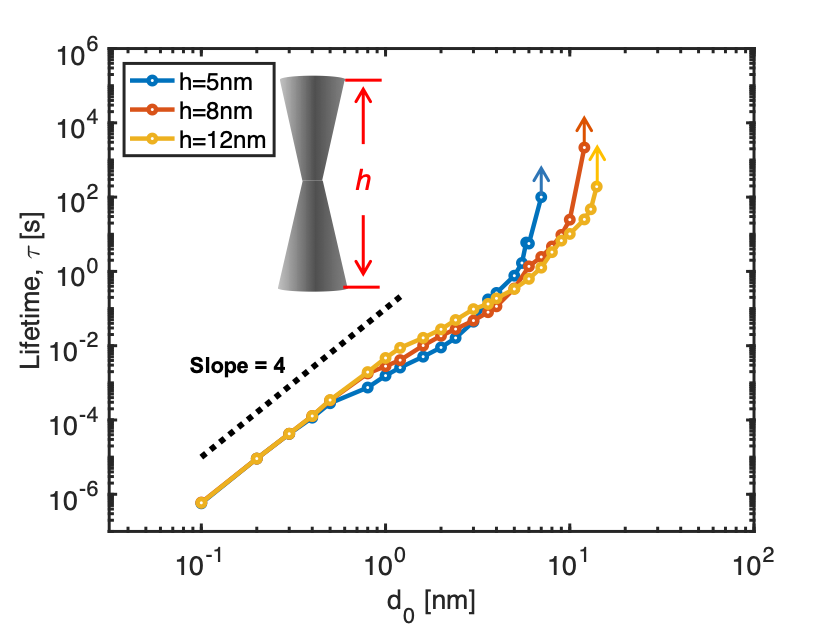


**Supplementary Figure 10.** **Filament lifetime as a function of d_0_ for various filament length.** Calculated filament lifetime for a conical shape with conical angle $\alpha=5^{\circ}$, as a function of the initial diameter for various filament length$h=5 \mathrm{nm}$, $h=8 \mathrm{nm}$, and$h=12 \mathrm{nm}$.

**
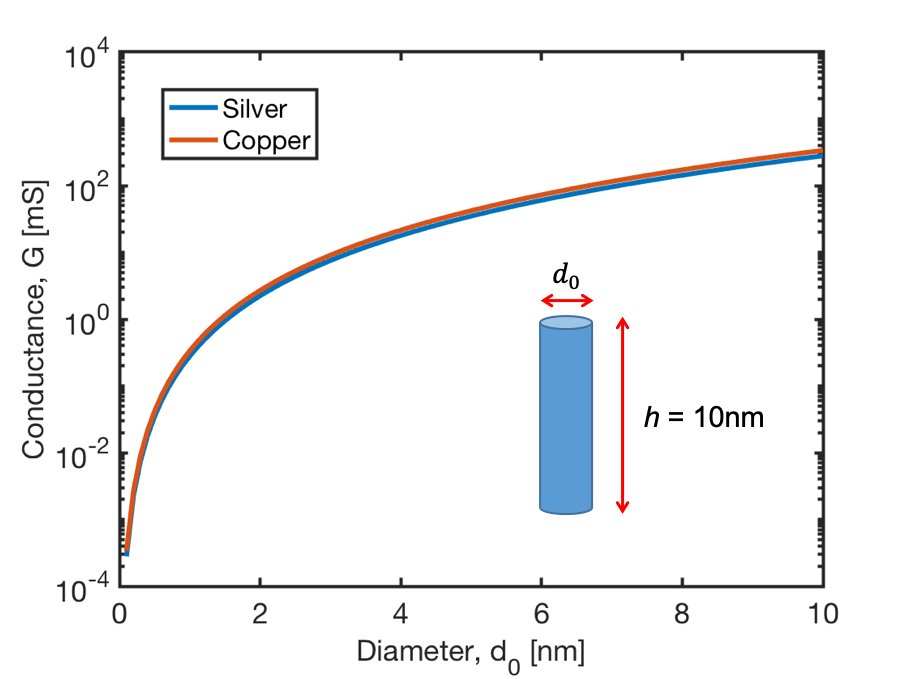
**

**Supplementary Figure 11.** Calculated conductance of the nanoscale filament as a function of the filament diameter. Surface scattering effect is included in the calculation.


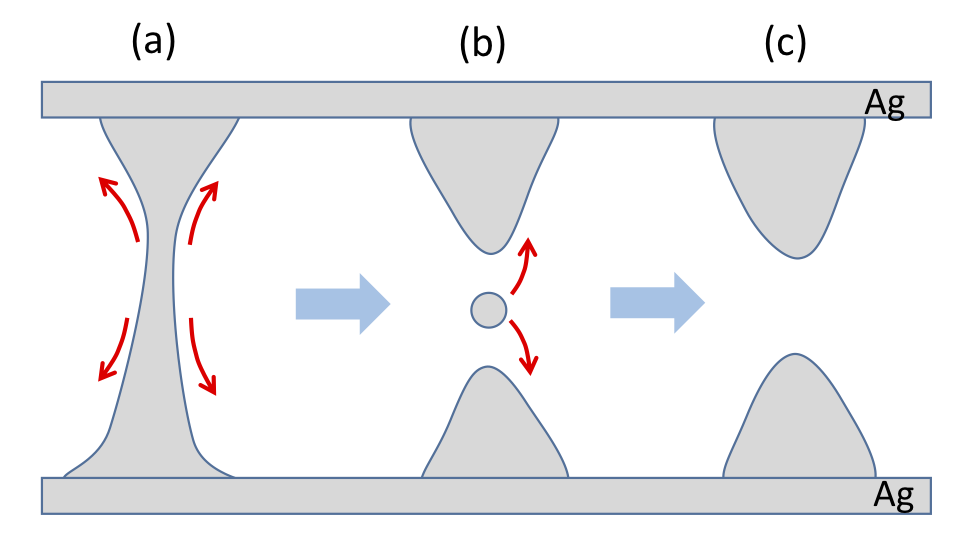


**Supplementary Figure 12.** **Surface diffusion and Ostwald ripening in volatile devices.** (a)-(b) Surface diffusion might controlled the initial stage of the filament disconnection determining the lifetime of the filament. (b)-(c) Post-lifetime evolution of the filament particle induced by Ostwald ripening.


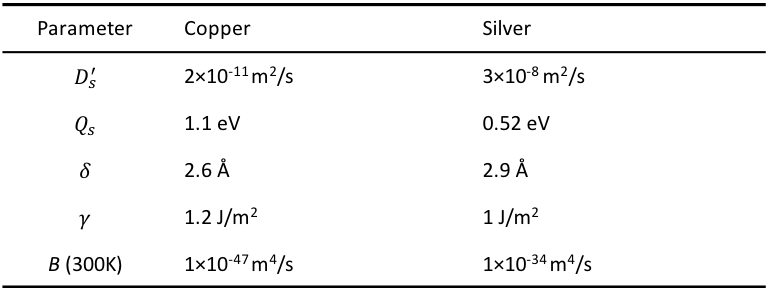


**Supplementary Table 1.** Parameters for the estimation of the values of *B* for copper and silver filaments.

**
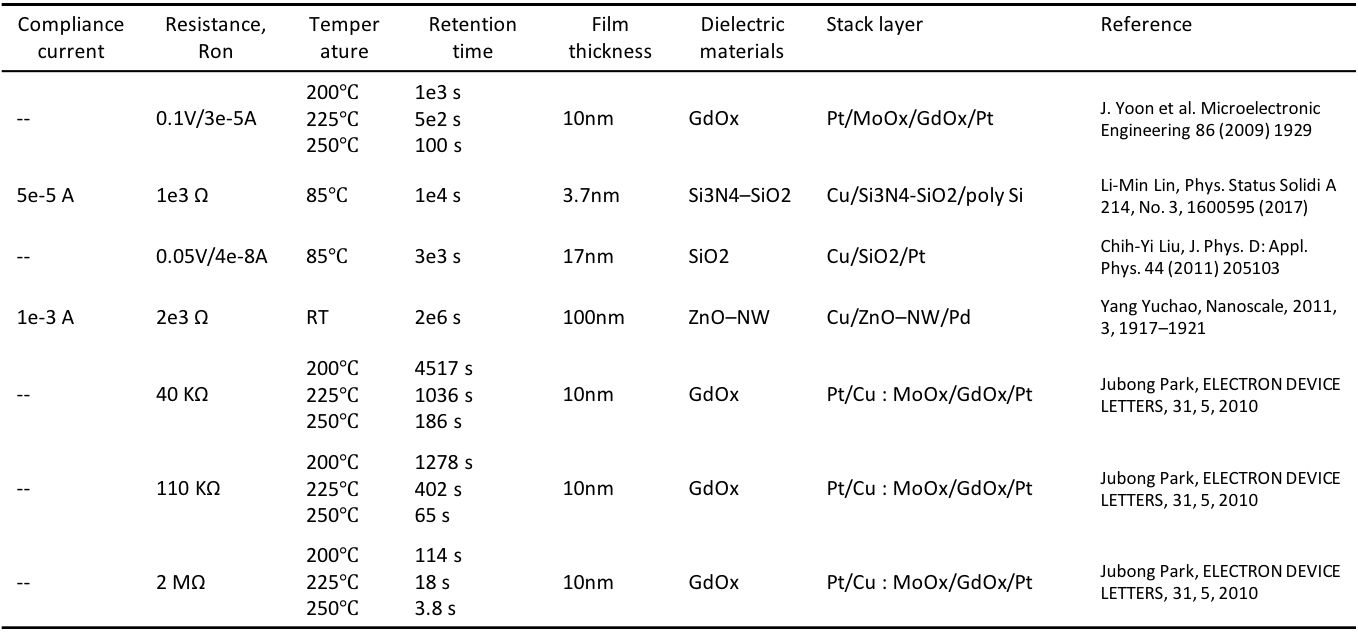
**

**Supplementary Table 2.** Data collection of the lifetime (retention time) for Cu filaments^1–5^.

**
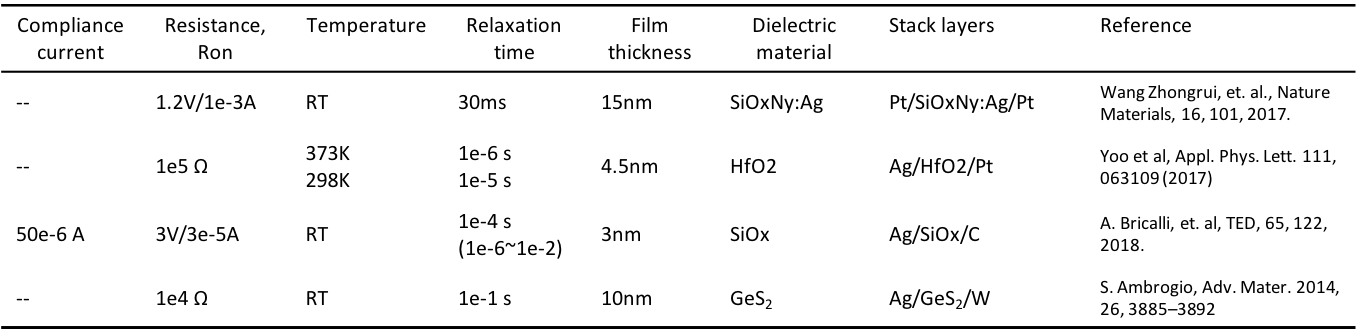
**

**Supplementary Table 3.** Data collection of the lifetime (relaxation time) for Ag filaments^6–9^.

**Supplementary Note 1.** **Bidirectional bipolar switching characteristics**

The device considered in our work has a symmetric structure with both electrodes being Ag NWs as shown in Fig. 1c. During set transition, the Ag atoms to form a filament come from the positively-biased electrode, since the Ag cations are migrating in the same direction as the electric field $F$. On the contrary, the reset transition takes place by applying a negative voltage on the same electrode. Taking as reference the voltage applied to the top electrode, the bipolar switching can thus take place by set transition under positive voltage and reset transition under negative voltage (Supplementary Fig. 1a, Supplementary Fig. 1c) or vice versa (Supplementary Fig. 1b and Supplementary Fig. 1d). The non-volatile switching shown in the figure occurs at high compliance current, hence a thick filament which is sufficiently stable to ensure non-volatile behavior.

**Supplementary Note 2.** **MD simulations of surface diffusion**

In this work, we explain the conductive filament (CF) morphological evolution by surface atom diffusion, which can be deduced from the MD simulation results in Fig. 2. Surface diffusion effects arise because surface atoms have larger free energy, and because diffusivity of the bulk atoms is limited due to their tendency to remain in the crystalline state. To further support the model of surface self-diffusion, we tracked the positions of individual atoms in the MD simulation, which is shown in Supplementary Fig. 2.

We first cut a horizontal slice of the filament in the top stub in the initial configuration, then we trace the position coordinate *z* of the sliced atoms as a function of time. Surface atoms quickly migrate toward the closer electrode, e.g., atoms 3875 and 3920 moving toward the top electrode (Supplementary Fig. 2a) and atoms 3680, 3729 and 3686 moving toward the bottom electrode (Supplementary Fig. 2b). On the other hand, bulk atoms remain in their positions although thermal fluctuations are observed, e.g., atoms 3890, 3905 in Supplementary Fig. 2a, and atoms 3705 and 3709 in Supplementary Fig. 2b. Atoms in the bottleneck (Supplementary Fig. 2c) randomly migrate toward either the top or the bottom electrode.

Fig. 2d and 2e summarize these simulation results by showing the calculated coordinate z for bulk atom A (3920) and surface atom B (3905) close to the top electrode, bulk atom C (3680) and surface atom D (3709) close to the bottom electrode, and bottleneck atoms E (3810) and F (3812).

The energy relaxation during the typical MD simulation of the evolution of the Ag filament (Fig. 2a-e) is shown in Supplementary Fig. 3, where the gradual decrease of the total energy is reported. From the initial state (near A) to the final state (C), the relaxation of the energy is about 65 eV (~ 10 aJ). This relaxation energy can be explained as the result of the energy minimization, and can give an estimate for the total energy change for the reverse process, namely the set transition. Note that, however, the real energy consumption for the switching operation should be much larger than this relaxation energy, since other energy expenses, such as the thermal dissipation needed to speed up the transition in a fast timescale, are needed.

**Supplementary Note 3.** **Details of the morphological evolution simulation**

The morphological evolutions of the filament geometry are simulated in dimensionless form^10^. We define $H=h/l$, $D_{0}=d_{0}/l$, $K=\kappa l$, $P=\rho/l$, $Z=z/l$, $S=s/l$, $N=n/l$ and $\Gamma=Bt/l^{4}$, where $h$ is the filament length (insulator thickness in the RRAM device), $d_{0}$ is the initial filament diameter, $\kappa$ is the surface curvature, $\rho$ defines the profile of the axisymmetric surface, $z$ is the axis of rotation, $s$ is the length of the profile $\rho$, $n$ is outward normal of the profile (Supplementary Fig. 4a), $t$ is time, and $H$, $K$, $P$, $Z$, $S$, $N$, $\Gamma$ are the corresponding dimensionless variables. $l$ (unit: $m$) is the scaling ratio between actual size of the filament geometry and the dimensionless one, defining the spatial dimension of the filament. Eq. (2) can thus be transformed into its dimensionless form,

$\frac{dN}{d\Gamma}=-\frac{1}{P}\frac{\partial}{\partial S}(P\frac{\partial K}{\partial S})$. (1)

Defining the angle $\theta$ between the tangent to the profile $\rho$ and the axis $z$ (Supplementary Fig. 4a), i.e., $tan\theta=\partial P/\partial Z$, the surface curvature can be written as,

$K=\frac{cos\theta}{P}-\frac{\partial\theta}{\partial S}$. (2)

The boundary conditions of the simulation are given by the contact angles between the filament and the electrodes, namely $\theta_{T}$ and $\theta_{B}$ in Supplementary Fig. 4b, which are related to the wettability of the materials in the filament, the electrodes and the insulating layer, similar to the contact angles of a liquid droplet on a solid surface. For instance, for the top boundary of the filament, the contact angle $\theta_{T}$ can be obtained from the Young Equation^11^,

$\gamma_{TE-I}-\gamma_{TE-F}+\gamma_{F-I}sin\theta_{T}=0$, (3)

where $\gamma_{TE-I}$ is the top electrode-insulator interfacial energy, $\gamma_{TE-F}$ is the top electrode-filament interfacial energy, and $\gamma_{F-I}$ is the filament-insulator interfacial energy. The range of the contact angle $\theta_{T}$ is from $-\pi/2$ to $\pi/2$, while the contact angle $\theta_{B}$ has opposite sign. A negative angle describes a hydrophobic effect with small electrode-filament interfacial $\gamma_{TE-F}$. In such case, the filament tends to detach from the top electrode, thus accounting for the lack of a stable filament in the upper right inset figure of Supplementary Fig. 8c. On the other hand, a positive $\theta_{T}$ indicates a high adhesive force between the top electrode and the filament material. Assuming the same materials for the filament and the electrodes, the contact angles should be $\pi/2$ for the top electrode and $-\pi/2$ for the bottom electrode. To provide a general illustration of the filament evaluation, we used boundary conditions of $\theta_{T}=\pi/6$ (for materials adhesive to silver, such as Pt^12^, Au, and graphitic carbon^8^) and $\theta_{B}=-\pi/2$ (same material as the filament), for the simulation results in Fig. 3.

In the simulation shown in Fig. 3, the initial filament geometries are assumed to be two reversed truncated cones, in which the conical angle is assumed to be $\alpha=5^{\circ}$ (Supplementary Fig. 5). Other possible initial shapes can be assumed, for instance, initial cylinder filament with $\alpha=0^{\circ}$. As $\alpha$ decreases, the filament lifetime decreases, without affecting the scaling rule of lifetime (see Supplementary Note 6). On the other hand, a different $\alpha$ results in a different morphological evolution of the filament, e.g., showing a larger tendency to nanoclustering for small $\alpha$ (Supplementary Movies 4-8).

To ensure the correctness of the simulation results, the volume change during the filament morphological evolution has been tracked. For the simulation results shown in Fig. 3e to Fig. 3j, the volume change was less than 1.5% (Supplementary Fig. 6), due to numerical errors in the simulation. Such a small error does not affect the validity of the simulation results.

**Supplementary Note 4.** **Estimation of the materials parameters of copper and silver**

The parameter $B=D_{s}\gamma\delta^{4}/kT$ is a geometry-independent parameter controlling the time normalization according to $t=\frac{\Gamma l^{4}}{B}$. The value of the parameter $B$ is strongly dependent on the filament material and the temperature, since the surface self-diffusion coefficient is governed by Arrhenius law $D_{s}=D_{s}^{'}exp(-\frac{Q_{s}}{kT})$, where the activation energy $Q_{s}$ is much larger than $kT$ at room temperature. The activation energy $Q_{s}$ for copper and silver were measured to be 1.6 $\mathrm{eV}$ and between 0.2 $\mathrm{eV}$ and 1.1 $\mathrm{eV}$, respectively^10,13^, from sphere sintering experiments at high temperature. Reporting the lifetimes of copper and silver filament on the Arrhenius plot provides estimates of $Q_{s}$ to be 1.3~1.4 $\mathrm{eV}$ and 0.5~0.7 $\mathrm{eV}$, respectively^5,7,9^. Varying the CF surrounding materials, e.g., HfO_x_ or SiO_x_, would change the interface condition, thus affecting the surface self-diffusion process. Different Ag/dielectric interfaces result in different values of the diffusion coefficient pre-factor $D_{s}^{'}$ and activation energy $Q_{s}$. To highlight the size-dependent lifetime behavior, we used fixed values of $D_{s}^{'}$ and $Q_{s}$ to estimate the values of *B* for copper and silver, respectively (Supplementary Table 1), for the calculation of the predictive lines shown in Fig. 5c. The deviations of the experimental data from the predictive lines can be regarded as the results of diffusion coefficient variation due to the different Ag/dielectric interfaces.

The value of the parameter $B$ is also affected by the surrounding materials, i.e., insulator materials, through the surface energy or surface tension $\gamma$. The values of surface energy for copper-vacuum and silver-vacuum surfaces are $1.2 J/m^{2}$ and $1 J/m^{2}$, respectively^14^. Considering the surrounding insulating material, the surface energy becomes^11^:

$\gamma=\gamma_{F}-\gamma_{I}+\gamma_{F-I}$, (4)

where $\gamma_{F}$ is the filament-vacuum surface energy, $\gamma_{I}$ is the insulator-vacuum surface energy, and $\gamma_{F-I}$ is the filament-insulator interfacial energy. In our experiment, the organic silk insulator possesses small values of $\gamma_{I}$ and $\gamma_{F-I}$, owing to its weak van der Waals interactions. Varying the insulating materials, e.g., HfO_x_ or SiO_x_, may result in a decrease of the surface energy, although the order of magnitude would remain the same. Therefore, the effect of the insulating material on the filament disruption time can be neglected with respect to the change of temperature $T$ and the scale of the filament $l$.

Supplementary Table 1 summarizes the parameters adopted in our study for copper and silver filaments, all being comparable to the values reported in the literature.

**Supplementary Note 5.** **Comparison between the MD simulation and the numerical model**

To validate the numerical simulation based on the surface diffusion model of Eq. (1) and Eq. (2), as an alternative to the MD simulation, we simulated the relaxation process for very similar initial shapes of the filament with the two models. The filament length is 10 nm, while the smallest filament diameter is $d_{0}=0.6 \mathrm{nm}$, with a conical angle of approximately $\alpha=10^{\circ}$. The filament shape evolution was computed according to the MD approach (Supplementary Fig. 7a) and the numerical model (Supplementary Fig. 7b). To compare the two models, the filament gap length as a function time is reported in Supplementary Fig. 7c for the two types of simulation. It should be noted that the parameters for the MD simulation were obtained from first principles and the simulation is assuming vacuum hosting environment with high temperature (800 K), while the parameters for the numerical model were obtained by fitting the filament lifetime to the experimental data. As a result, the timescales for the two models are not consistent, thus a normalized time is used in the comparison of Supplementary Fig. 7c. The simulation of the filament shape evaluation was collected as a function of time in the Supplementary Movie 3 to better highlight the similar dynamics in the simulated relaxation.

**Supplementary Note 6.** **Impact of the electrode material, initial filament shape and filament length**

Supplementary Fig. 8a shows the calculated CF lifetime for $\theta_{T}=\pi/3$ and $\theta_{B}=-\pi/2$, while Supplementary Fig. 8b shows the calculated CF lifetime for $\theta_{T}=\pi/2$ and $\theta_{B}=-\pi/2$, i.e., with higher top electrode wettability compared to (a). Simulation results show that different boundary conditions due to a change of the electrode materials does not significantly affect the size dependence of the lifetime. For comparison, the stable capillary bridge is formed for $d_{0}=11 \mathrm{nm}$ in Supplementary Fig. 8a and Supplementary Fig. 8b, compared to $d_{0}=14 \mathrm{nm}$ in Fig. 4. On the other hand, if the top electrode is hydrophobic with respect to the filament, no stable capillary bridge can be formed, thus the lifetime follows Herring’s law across the whole range of initial diameter (Supplementary Fig. 8c and Supplementary Fig. 8d).

We also calculated the filament lifetime for various conical angle (Supplementary Fig. 9) of the initial filament to study its impact on the filament disconnection dynamics. Results in Supplementary Fig. 9 indicate that the lifetime is negligibly impacted by the initial filament shape. A cylindrical filament ($\alpha=0^{\circ}$) shows a slightly shorter lifetime than the conical shapes ($\alpha>0^{\circ}$), which is due to i) the highest curvature being located in correspondence with the top and bottom electrodes with an angle of π/2, compared to a larger angle π/2 – α for the conical shape; ii) zero vertical principle curvature compared to a negative vertical principle curvature in the bottleneck for the conical shape. On the other hand, the lifetime is still strongly controlled by the initial filament diameter. The shape also controls the type of evolution of the filament, for instance a thin cylindrical filament evolves into a sequence of nano-spheres dispersed within the dielectric layer (Supplementary Movie 7), whereas a conical shape is generally disconnected at the bottleneck and ends up in two stubs contacting the top and bottom electrodes, respectively (Supplementary Movie 4).

For a given initial filament diameter, the filament length plays a negligible role in the filament lifetime as shown in Supplementary Fig. 10. This can be explained by the filament disconnection taking place in correspondence of the bottleneck of the filament, while the distance of the bottleneck from the electrodes plays a minor role, at least for $h>>d_{0}$. The filament length only plays a non-negligible role in the critical $d_{0}$ controlling the transition from volatile to nonvolatile behavior, because of the limited migration distance available for relatively short filaments compared to long filaments. However, this difference is seen for relatively large values of $d_{0}$ in the range around and above 10 nm, which is larger than the experimental range shown in Fig. 5c.

**Supplementary Note 7.** **Surface self-diffusion and out-diffusion**

Besides the surface self-diffusion mechanism, another possible mechanism, namely volume diffusion or out-diffusion, has been proposed for the spontaneous rupture of the conductive filament in resistive switching device^15^. However, according to recent TEM observations and the analysis shown in this article, in the considered material system, i.e., Ag and Cu in oxides or polymer host materials, we believe that surface diffusion, rather than out-diffusion, dominates the filament shape evolution and lifetime.

In recent high resolution TEM observations, the Ag or Cu clusters other than homogeneous Ag or Cu doped dielectric layers are often observed^6,12,16,17^. The material systems are Ag in SiO_x_ (x<2)^6^, Cu in SiO_x_ (x<2)^16^, and Ag in SiO_2_^12,17^, respectively, all indicating low solubilities of Ag and Cu in silicon oxide and the tendency of Ag or Cu atoms to cluster together. Particularly, in ref.^16^, the device was fabricated with Ag doping in SiO_x_, however, the as-fabricated device shows Ag clusters in SiO_x_ other than homogeneous Ag doped in SiO_x_ layer in the TEM image. From the perspective of electrochemistry, the low solubility of Ag or Cu in such host materials can be considered as the results of: i) Ag and Cu are generally inert metal and hard to be oxidized (for instance, in metal reactivity series^18^: Al > Ti >Cr > Ni > Pb > Cu > W > Ag > Au; electronegativity (Allen scale)^19^: Hf (1.16) < Ta (1.34) < Ti (1.38) < W (1.47) < Cu (1.85) < Ag (1.87) < Au (1.92)); ii) the strong chemical stability of Si-O valence bond. In our experiments, we used polymer (silk) as dielectric layer between two Ag NW electrodes. Ag atoms are more likely to cluster together rather than diffuse as single atoms, given the low affinity of the hosting polymer with the metal atom. In the calculation-data comparison, the filament lifetime data are all collected from devices with dielectric layer of active-metal oxide (SiO_x_, GdO_x_, HfO_2_, etc.).

Out-diffusion might play a non-negligible role in other materials systems, such as when the metallic element (Ag, Cu) has a high solubility in the host materials, e.g., metal-chalcogenide materials, or more specifically, Ag_2_S for Ag filament^20^, or CuS for Cu filament^21^. In these cases, a somewhat different analysis should be conducted. However, in our experiments, we focused on polymer (silk) as dielectric layer between two Ag NW electrodes. Ag atoms are more likely to cluster together rather than diffuse homogeneously as single atoms, given the low solubility of Ag into the host polymer. As a result, silk almost behaves as vacuum in this case, which is what has been simulated in Fig. 2. Similarly, all data collected in Fig. 5 refer to oxide dielectric layers, such as SiO_x_^6,12,16,17^, GdO_x_^1,5^, HfO_2_^7^, and others, which all share a low solubility with the active metals Ag or Cu. The relative solubility of Ag in SiO_2_ is 10^-6^, namely three orders of magnitude smaller than in Ag_2_S beta phase where the Ag solubility is 10^-3^ at room temperature (300 ℃)^22,23^. Thus, our main finding about the leading role of surface diffusion in controlling the lifetime of RRAM should be restricted to these material systems (polymers, oxides) with relatively-low solubility of Ag, rather than solid electrolyte materials with relatively-high solubility.

Assuming out-diffusion mechanism, on the other hand, would result in a smaller exponent of the lifetime scaling rule^15,24^,

$s_{\tau}\sim\lambda^{2}$, (5)

where $s_{\tau}$ and $\lambda$ are the scaling factors of time and dimension, respectively, for geometrically identical changes. Supplementary Eq. (5) is a result of the second-order differential in the Fick’s second law. The smaller exponent of the scaling law cannot account for the strong size dependence of filament lifetime in Fig. 5.

**Supplementary Note 8.** **The Herring’s scaling law**

For the mechanism of surface-tension driven self-diffusion, C. Herring^25^ predicted the scaling law of the lifetime for identical geometrical changes except for a difference of scale, namely $s_{\tau}\sim\lambda^{4}$, where $s_{\tau}$ is the scaling factor of the time required for the identical geometrical change, and $\lambda$ is the scaling factor of size. Herring’s law was derived from the simple observation that the size scaling by a factor $\lambda$ results in the volume of material to be transported scaling by $\lambda^{3}$ and the mass flux scaling by ${1/\lambda}^{2}$ (also as the result of Eq. (1)). The total rate of the transport is the product of the flux by a length in the surface and normal to the flux direction, thus yielding:

$s_{\tau}\sim\frac{(amount)}{(rate)}\sim\frac{(amount)}{\left( flux \right)\lambda}\sim\frac{\lambda^{3}}{1/\lambda^{2}\cdot\lambda}=\lambda^{4}$. (6)

Herring’s law can also be obtained from Eq. (2) or Supplementary Eq. (1), describing the morphological change rate as a fourth-order differential of the filament dimension. In Eq. (2), the filament surface evolution rate $dn/dt$ is proportional to the second-order derivative of the curvature *κ*, which in turn is a second-order derivative of the surface profile $\rho$ in Eq. (1).

**Supplementary Note 9.** **Conductance of the nanoscale filament**

To compare the predicted lifetime with the measured data, the relationship between conductance and the size of the metallic filament needs to be clarified. The filament resistance is given by $R=4h/(\pi\sigma d_{0}^{2})$, where $\sigma$ is the conductivity of the constituent metal in the CF. From this relationship, one can estimate the initial diameter d_0_ of the filament from the measured device resistance $R$. When the size of the filament is much smaller than the length of electron free path, $\sigma$ can be given by^26,27^,

$\sigma=\sigma_{0}\frac{1+p}{1-p}\frac{d}{l_{f}}$, (7)

where $\sigma_{0}$ is the bulk conductivity of the metal, $p$ is the fraction of electrons scattered at the surface, $d$ the width of the wire, and $l_{f}$ the mean free path of the bulk material. The values of the bulk resistivity $1/\sigma_{0}$ are $1.75 \mu\Omega\cdot cm$ and $1.59 \mu\Omega\cdot cm$ for copper and silver, respectively. The electron mean free path $l_{f}$ is $40 \mathrm{nm}$ and $53 nm$ for copper and silver^28^, respectively, while $p=0.5$ can be reasonably assumed. Supplementary Fig. 11 shows the calculated conductance of the copper and silver filament with cylinder shape and height of 10 nm as a function of the diameter.

When the filament is disrupted (for the calculation of the conductance of the device in high resistance state in Fig. 5b), the conductance is simply calculated by only accounting the conductance of the gap between the filament segments, $G=\sigma_{I}A/g$, where $\sigma_{I}$ is the conductivity of the insulator material, $A$ is the effective area of the leakage channel between to discontinued filament segments, and $g$ is the gap length.

**Supplementary Note 10.** **Gibbs-Thomson effect and Ostwald ripening**

Gibbs-Thomson effect has several equivalent forms, one of which is that the vapor pressure of liquid-vapor interface is proportional to its radius. The vapor pressure $p$ for a curved interface with principal radii $r_{1}$ and $r_{2}$ can be given by^14,29^,

$p=p_{0}-\frac{\gamma\rho_{v}}{\rho_{l}-\rho_{v}}(\frac{1}{r_{1}}+\frac{1}{r_{2}})$, (8)

where $p_{0}$ is the vapor pressure for a flat interface ($r_{1},r_{2}=\infty$), $\gamma$ the surface tension, $\rho_{v}$ the density of vapor, and $\rho_{l}$ the density of liquid. It can be extended to nanoparticle surface, which describes the surface concentration $C$ of the particle atoms by^30^,

$C=C_{0}exp(\frac{2\gamma\Omega}{kTr})$, (9)

where $r$ is the particle radius, $C_{0}$ is the surface concentration of atoms in an infinitely large particle, $\Omega$ is the atomic volume, $k$ is the Boltzmann constant and $T$ is the temperature. For instance, Supplementary Eq. (9) can be used to describe Ostwald ripening of nanoparticles in the solid state.^31,32^

Note that Supplementary Eq. (8) and Supplementary Eq. (9) are somehow equivalent, as they both predict that larger particles grow at the expense of smaller ones. The reason is that the surface of smaller particle has larger pressure (Supplementary Eq. (8)) or higher concentration (Supplementary Eq. (9)) of the particle atoms. However, neither Supplementary Eq. (8) nor Supplementary Eq. (9) can be used to directly predict the surface diffusion phenomenon and the evolution of the filament shape, which is instead obtained by Eq. (1) and Eq. (2) in our numerical model. Yet, a key term in Eq. (1) and Eq. (2) is the sum of inverse principal radii, similar to Supplementary Eq. (8), which highlights that the underlying physics in out surface diffusion model still has roots in the Gibbs-Thomson effect.

In our investigated material systems, Ag or Cu filament within silk, or SiO_2_, the main physical process driving the filament shape evolution and controlling the device lifetime is the surface diffusion, rather than out-diffusion as in Ostwald ripening. This is because atomic movement at the surface (or interface) is easier than movement of atoms across the surrounding host material. While out-diffusion is essential to describe Ostwald ripening of separate particles^31,32^, surface diffusion is the more direct mechanism to minimize the surface-volume ratio within a single filament, or when particles are touching one to each other^14,33^. The Ostwald ripening has been proposed to control the evolution of the particles obtained by filament fragmentation^6^. The surface diffusion controls the initial stages of the filament disconnection (Supplementary Fig. 12a-b), which also dictates the filament lifetime according to the Herring’s law. Ostwald ripening instead may be responsible for the post-lifetime evolution of the filament particles (Supplementary Fig. 12b-c). The driving force for the surface diffusion and Ostwald ripening both can be traced back to the Gibbs-Thomson effect.

**Supplementary Note 11.** **Endurance of the silver/copper filament-based devices**

The RRAM devices can be operated in either the volatile or the nonvolatile regime, just by changing the compliance current *I*_C_. In the volatile regime, the operation just require that a voltage pulse, e.g., with positive voltage, is applied to induce the transition to the on state, from which the device automatically relaxes to the off state after a stochastic retention time. For relatively high compliance, the device remains in the on state after switching, which requires the application of another pulse with reversed polarity to induce the reset transition to the off state.

While the retention strongly depends on the type of switching, i.e. either volatile or nonvolatile, the endurance does not show any obvious dependence on the type of volatile/nonvolatile switching. For instance, a high endurance *N*_C_ was reported for several volatile RRAM devices, such as *N*_C_ = 10^8^ for Pd/Ag/HfO_x_/Ag/Pd^34^, *N*_C_ = 10^6^ for AgNW/silk/AgNW^35^, *N*_C_ = 10^6^ for Pt/SiO_x_N_y_:Ag/Pt^6^, and *N*_C_ = 10^10^ for Cu/HfO_2_:Cu/Pt^36^. On the other hand, comparably high endurance was reported for nonvolatile RRAM devices, such as *N*_C_ = 10^9^ for Cu/TaO_x_/Pt^37^ and *N*_C_ = 10^6^ for CuTe_x_/Al_2_O_3_/TiN^38^.

Since the same device can be operated in volatile or nonvolatile mode just by changing the compliance current *I*_C_, one may wonder whether the device endurance is affected by *I*_C_. Although there are currently no data comparing endurance on the same device operated in volatile mode (low *I*_C_) or nonvolatile mode (high *I*_C_), previous data on nonvolatile RRAM devices with HfO_x_ dielectric indicate no dependence of endurance on *I*_C_^39^. This was explained by the fact that a larger *I*_C_ is accompanied by a larger filament area, while the current density, which controls the local Joule heating and associated device/material degradation, remains constant. From these results, there might be no dependence of endurance on the volatile/nonvolatile switching mode.

**Supplementary References**

1. Yoon, J. *et al.* Analysis of copper ion filaments and retention of dual-layered devices for resistance random access memory applications. *Microelectron. Eng.* **86,** 1929–1932 (2009).

2. Lin, L. M. *et al.* A novel nanoscale-crossbar resistive switching memory using a copper chemical displacement technique. *Phys. Status Solidi Appl. Mater. Sci.* **214,** 1600595 (2017).

3. Liu, C.-Y., Huang, Y.-H., Ho, J.-Y. & Huang, C.-C. Retention mechanism of Cu-doped SiO_2_-based resistive memory. *J. Phys. D. Appl. Phys.* **44,** 205103 (2011).

4. Yang, Y. *et al.* Nonvolatile resistive switching in single crystalline ZnO nanowires. *Nanoscale* **3,** 1917 (2011).

5. Park, J. *et al.* Investigation of state stability of low-resistance state in resistive memory. *IEEE Electron Device Lett.* **31,** 485–487 (2010).

6. Wang, Z. *et al.* Memristors with diffusive dynamics as synaptic emulators for neuromorphic computing. *Nat. Mater.* **16,** 101–108 (2017).

7. Yoo, J., Park, J., Song, J., Lim, S. & Hwang, H. Field-induced nucleation in threshold switching characteristics of electrochemical metallization devices. *Appl. Phys. Lett.* **111,** 063109 (2017).

8. Bricalli, A. *et al.* Resistive switching device technology based on silicon oxide for improved on-off ratio – part II: select devices. *IEEE Trans. Electron Devices* **65,** 122–128 (2018).

9. Ambrogio, S., Balatti, S., Choi, S. & Ielmini, D. Impact of the mechanical stress on switching characteristics of electrochemical resistive memory. *Adv. Mater.* **26,** 3885–3892 (2014).

10. Nichols, F. A. & Mullins, W. W. Morphological changes of a surface of revolution due to capillarity-induced surface diffusion. *J. Appl. Phys.* **36,** 1826–1835 (1965).

11. Finnis, M. W. The theory of metal - ceramic interfaces. *J. Phys. Condens. Matter* **8,** 5811–5836 (1996).

12. Zhao, X. *et al.* Breaking the current-retention dilemma in cation-based resistive switching devices utilizing graphene with controlled defects. *Adv. Mater.* **30,** 1705193 (2018).

13. Rhead, G. E. Surface self-diffusion of silver in various atmospheres . *Acta Metall.*  **13,** 223–226 (1965).

14. Kuczynski, G. C. Self-diffusion in sintering of metallic particles. *JOM* **1,** 169–178 (1949).

15. Aga, F. G. *et al.* Retention modeling for ultra-thin density of Cu-based conductive bridge random access memory (CBRAM). *AIP Adv.* **6,** 025203 (2016).

16. Yuan, F. *et al.* Real-time observation of the electrode-size-dependent evolution dynamics of the conducting filaments in a SiO_2_ layer. *ACS Nano* **11,** 4097–4104 (2017).

17. Yang, Y. *et al.* Electrochemical dynamics of nanoscale metallic inclusions in dielectrics. *Nat. Commun.* **5,** 4232 (2014).

18. Greenwood, N. N. (Norman N. & Earnshaw, A. (Alan). *Chemistry of the elements*. (Pergamon Press, 1984).

19. Mann, J. B., Meek, T. L. & Allen, L. C. Configuration Energies of the Main Group Elements. *J. Am. Chem. Soc.* **122,** 2780–2783 (2000).

20. Ohno, T. *et al.* Short-term plasticity and long-term potentiation mimicked in single inorganic synapses. *Nat. Mater.* **10,** 591–595 (2011).

21. Sakamoto, T. *et al.* Nanometer-scale switches using copper sulfide. *Appl. Phys. Lett.* **82,** 3032–3034 (2003).

22. McBrayer, J. D., Swanson, R. M. & Sigmon, T. W. Diffusion of Metals in Silicon Dioxide. *J. Electrochem. Soc.* **133,** 1242 (1986).

23. Sharma, R. C. & Chang, Y. A. The Ag−S (Silver-Sulfur) system. *Bull. Alloy Phase Diagrams* **7,** 263–269 (1986).

24. Ielmini, D., Nardi, F., Cagli, C. & Lacaita, A. L. Size-dependent retention time in NiO-based resistive-switching memories. *IEEE Electron Device Lett.* **31,** 353–355 (2010).

25. Herring, C. Effect of change of scale on sintering phenomena. *J. Appl. Phys.* **21,** 301–303 (1950).

26. Steinhögl, W., Schindler, G., Steinlesberger, G. & Engelhardt, M. Size-dependent resistivity of metallic wires in the mesoscopic range. *Phys. Rev. B* **66,** 075414 (2002).

27. Sondheimer, E. H. The mean free path of electrons in metals. *Adv. Phys.* **1,** 1–42 (1952).

28. Gall, D. Electron mean free path in elemental metals. *J. Appl. Phys.* **119,** 085101 (2016).

29. Thomson, W. LX. On the equilibrium of vapour at a curved surface of liquid. *London, Edinburgh, Dublin Philos. Mag. J. Sci.* **42,** 448–452 (1871).

30. Simonsen, S. B. *et al.* Ostwald ripening in a Pt/SiO2 model catalyst studied by in situ TEM. *J. Catal.* **281,** 147–155 (2011).

31. Simo, A. *et al.* Formation mechanism of silver nanoparticles stabilized in glassy matrices. *J. Am. Chem. Soc.* **134,** 18824–18833 (2012).

32. Simonsen, S. B. *et al.* Direct Observations of Oxygen-induced Platinum Nanoparticle Ripening Studied by In Situ TEM. *J. Am. Chem. Soc.* **132,** 7968–7975 (2010).

33. Mullins, W. W. Theory of thermal grooving. *J. Appl. Phys.* **28,** 333–339 (1957).

34. Midya, R. *et al.* Anatomy of Ag/Hafnia-based selectors with 10^10^ nonlinearity. *Adv. Mater.* **29,** 1604457 (2017).

35. Wang, M. *et al.* Enhancing the Matrix Addressing of Flexible Sensory Arrays by a Highly Nonlinear Threshold Switch. *Adv. Mater.* **30,** 1802516 (2018).

36. Luo, Q. *et al.* Cu BEOL compatible selector with high selectivity (>10^7^), extremely low off-current (~pA) and high endurance (>10^10^). in *2015 IEEE International Electron Devices Meeting (IEDM)* 10.4.1-10.4.4 (IEEE, 2015). doi:10.1109/IEDM.2015.7409669

37. Lv, H. *et al.* Evolution of conductive filament and its impact on reliability issues in oxide-electrolyte based resistive random access memory. *Sci. Rep.* **5,** 7764 (2015).

38. Robayo, D. A. *et al.* Statistical analysis of CBRAM endurance. in *2018 International Symposium on VLSI Technology, Systems and Application (VLSI-TSA)* **1,** 1–2 (IEEE, 2018).

39. Balatti, S. *et al.* Voltage-Controlled Cycling Endurance of HfOx-Based Resistive-Switching Memory. *IEEE Trans. Electron Devices* **62,** 3365–3372 (2015).
