# Supplementary material for: Surface diffusion-limited lifetime of silver and copper nanofilaments in resistive switching devices
Source: Nat Commun. 2019 Jan 8;10:81. doi: 10.1038/s41467-018-07979-0 (PMC6325242; doi:10.1038/s41467-018-07979-0)
Supplement: Supplementary file 3 — Description of Additional Supplementary Files [file 41467_2018_7979_MOESM3_ESM.pdf]

Description of Additional Supplementary Files

Supplementary Movie 1:

MD simulation of silver filament relaxation (shape 1, T = 800 K, 500 ps).

Supplementary Movie 2:

MD simulation of silver filament relaxation (shape 2, T = 800 K, 500 ps).

Supplementary Movie 3:

Comparing MD and numerical simulations.

Supplementary Movie 4:

Filament morphological evolution, with initial diameter 0.4 nm and conical angle 5°.

Supplementary Movie 5:

Filament morphological evolution, with initial diameter 2 nm and conical angle 5°.

Supplementary Movie 6:

Filament morphological evolution, with initial diameter 10 nm and conical angle 5°.

Supplementary Movie 7:

Filament morphological evolution, with initial cylinder filament and diameter 0.4 nm.

Supplementary Movie 8:

Filament morphological evolution, with initial cylinder filament and diameter 2 nm.
